# Supplementary material for: A ubiquitin-like protein encoded by the “noncoding” RNA TINCR promotes keratinocyte proliferation and wound healing
Source: PLoS Genet. 2021 Aug 5;17(8):e1009686. doi: 10.1371/journal.pgen.1009686 (PMC8341662; doi:10.1371/journal.pgen.1009686)
Supplement: Attachment — Submitted filename: nita_resp_pgrev2.doc [file pgen.1009686.s008.doc]

**Response to Reviewer #1**

We thank the reviewer for the careful evaluation of our manuscript and for the statements that “This result is of broad interest and important for researchers in the growing field of lncRNA biology” and that “The manuscript satisfies Plos Genetics’ citeria of originality, importance to researchers in the field, and broad interest to researchers in genetics and genomics.” We also thank the reviewer for constructive suggestions that we feel have helped us to greatly improve our manuscript.

Our specific responses to the points raised are as follows:

*The main open question is, by which mechanism does ULCAR accelerate the cell cycle / promote keratinocyte proliferation / wound healing? Full elucidation of the mechanism may be out of scope of this study, but mechanistic evidence and conclusions should match.*

*As the mechanism of cell cycle acceleration is uncertain and it is not known if this is the main role of the protein, the proposed name "Ubiquitin-Like Cell cycle AcceleratoR (ULCAR)" remains to be accepted by the research community. "ULCAR" should not be part of the title. The title is self-contradictory (noncoding / encodes) and should be changed from*

*"The long noncoding RNA TINCR encodes a ubiquitin-like protein (ULCAR) that promotes keratinocyte proliferation and wound healing" to "A ubiquitin-like protein encoded by the reportedly “noncoding“ RNA TINCR promotes keratinocyte proliferation and wound healing"*

**[Response]** We agree with the reviewer that it remains unclear whether ULCAR directly regulates the cell cycle, and we have therefore now renamed the protein TUBL, for TINCR-encoded ubiquitin-like protein. We also changed the title to “A ubiquitin-like protein encoded by the “noncoding” RNA TINCR promotes keratinocyte proliferation and wound healing.”

To explore the molecular function of TUBL, we purified protein complexes containing TUBL from NHEKs and HaCaT cells transiently expressing TUBL-(C)FLAG by immunoprecipitation with antibodies to FLAG. The eluted proteins were subjected to semiquantitative LC-MS/MS analysis, resulting in the identification of several molecules that were specifically associated with TUBL on the basis either that they were detected specifically in at least one TUBL-(C)FLAG replicate or that the average iBAQ (intensity-based absolute quantification) value for the three TUBL-(C)FLAG replicates was >2 times that for the control replicates (**new Figure 7A and new S3 Table**). Gene ontology (GO) term analysis of the identified TUBL binding proteins revealed enrichment of several cell cycle–related terms, such as those involving “ubiquitin-protein ligase activity involved in mitotic cell cycle” (**new Figure 7B**). However, we found that most genes constituting such GO terms actually encoded subunits of the proteasome complex, with many being commonly identified in both HaCaT and NHEK cells (**new Figure 7C and new S4 Table**).

TUBL is a type 2 Ubl protein but does not harbor any other apparent domains. It is therefore a Ubl-only protein that associates with the proteasome. In contrast, RAD23 and DSK2, both of which are representative type 2 Ubl proteins, contain a Ubl domain that associates with the proteasome and a Uba (ubiquitin-associated) domain that interacts with ubiquitylated proteins, allowing them to facilitate the recruitment of ubiquitylated proteins to the proteasome for efficient proteolysis. TUBL is expected to lack such an ability as a result of its lack of a Uba domain, suggesting that TUBL might function as a negative regulator that modifies the interaction between authentic Ubl-Uba proteins and the proteasome. Although the detailed molecular mechanism by which TUBL regulates cell cycle progression remains unknown, we believe that it is beyond the scope of our present study. We have now addressed these issues in the revised manuscript (page 11, lines 282-305).

*Lines 193-195: the following sentence should be extended by stating that expression in proliferating keratinocytes is lower than in total epidermis – suggesting that expression in differentiated keratinocytes is very significant, "TINCR, and found that it was expressed not only in differentiated keratinocytes but also in actively proliferating primary mouse keratinocytes to a greater extent than in other adult mouse tissues (Fig 3F),"*

**[Response]** As suggested, we have modified the text in the revised manuscript (page 9, lines 225-228).

*Can the authors exclude or confirm that TINCR / ULCAR is expressed in hair follicles? Might its deletion affect the hair cycle and indirectly influence wound healing?*

**[Response]** The skin darkens as a result of melanin deposition in hair follicles when the hair cycle transitions from telogen to anagen. In the wound healing experiment shown in Figure 6, we extensively removed hairs on the back of the mice, but the darkened skin area at 10 days after hair removal did not differ between *Tubl*^+/+^ and *Tubl*^−/−^ mice (**Figure R1 for reviewing purposes only**), suggesting that TUBL deficiency is unlikely to affect the hair cycle.

**Response to Reviewer #2**

We thank Dr. Pagano for the careful evaluation of our manuscript and for the statement that “The paper is a wonderfully clear and concise description of a real breakthrough, and the experiments are presented in a straightforward and logical fashion.” This reviewer has no suggestions for changes to the paper.

**Response to Reviewer #3**

We thank the reviewer for the careful evaluation of our manuscript and for the statement that “This is a very exciting and novel study showing a new protein the authors termed as ULCAR was identified with the lncRNA TINCR.” We also thank the reviewer for suggestions that we feel have helped us to greatly improve our manuscript.

Our specific responses to the points raised are as follows:

Major concerns:

*1. To distinguish if the observed effects are derived from ULCAR protein function or TINCR RNA function-A good control I would suggest is to generate siRNA/shRNA/sgRNA targeting TINCR but not ULCAR, or both, and examine effects of these loss-of-function experiments.*

**[Response]** Given that TUBL (we renamed ULCAR as TUBL, for TINCR-encoded ubiquitin-like protein, in response to a comment from another reviewer) is encoded by TINCR, it is technically difficult to attenuate the expression of TINCR without affecting that of TUBL. We therefore depleted both TINCR and TUBL by siRNA transfection in NHEKs, and found that the proportion of cells in S phase of the cell cycle was markedly decreased in the cells transfected with siRNAs targeting both TINCR and TUBL compared with those transfected with a control siRNA (**new Figure 2H**), similar to the results obtained with primary keratinocytes derived from TUBL-deficient and control mice. These data further support the conclusion that TUBL promotes keratinocyte proliferation. We have now addressed this point in the revised manuscript (page 7, lines 179-182).

*2. The authors mentioned that in multiple cancers the abundance of TINCR is up-regulated and UCLAR negatively regulates TINCR- does TINCR also regulate ULCAR levels?*

**[Response]** To investigate whether TINCR affects the expression level of TUBL protein, we transiently transfected HEK293T cells with vectors both for TUBL-(C)FLAG (ORF region only) and for an almost full-length form of TINCR that does not produce TUBL as a result of the introduction of a 1-bp deletion (WT_full_del). We found that the expression level of TUBL was not altered in the presence of WT_full_del (**new Figure 3F**), suggesting that TINCR is unlikely to contribute to the regulation of TUBL protein expression. We have clarified this point in the revised manuscript (page 8, lines 207-213).

Minor concerns:

*1. Fig. 1F: It seems that an N-terminal V5 tag abolishes ULCAR expression-can the authors provide some explanations?*

**[Response]** Addition of the NH_2_-terminal V5 tag resulted in a decrease in the expression of mouse TUBL, but not that of human TUBL (**Figure 1F**). Unlike that of human and other species, mouse TUBL has two potential AUG initiation codons (**Figure 1B**), and we inserted the V5 tag immediately after the second AUG initiation codon (**Figure 1F**), which might have impaired translation initiation. In addition, given that small proteins are often destabilized by the addition of tags (our unpublished results), the addition of the V5 tag to the NH_2_-terminus may have adversely affected the stability of mouse TUBL specifically by an unknown mechanism. We have now addressed this issue in the revised manuscript (pages 5-6, lines 136-142).

*2. Fig. 1G: The Flag signal was not observed in input but in IPs- can the authors provide some explanations?*

**[Response]** This is probably because of the low expression level of TUBL. We had used the epidermis of *Tubl*^FLAG/+^ (heterozygous) mice to examine the expression of TUBL-FLAG in the original manuscript. In the revised manuscript, however, we used the epidermis of *Tubl*^FLAG/FLAG^ (homozygous) mice so as to increase the amount of the tagged protein. As expected, we were then able to detect TUBL-FLAG in the input (**new Figure 1G**) We have now addressed these points in the revised manuscript (page 6, lines 143-148).

*3. Fig. 3: it is an elegant design to generate proposed mutants to disrupt either RNA or protein function- however, assays performed in this figure rely on overexpression conditions. The presence of endogenous TINCR and ULCAR may complicate the interpretation of data in this figure.*

**[Response]** In response to this comment, we set out to examine the effects of TUBL overexpression in cells depleted of endogenous TINCR or TUBL. We thus tried to monitor BrdU uptake in NHEKs both overexpressing TUBL (ORF region only) and transfected with siRNAs targeting both TINCR and TUBL. Unfortunately, even the control cells did not incorporate BrdU sufficiently, probably as a result of stress induced by the simultaneous lentivirus infection and siRNA treatment. Although we were unable to conduct the experiment suggested by the reviewer, consistent effects on the cell cycle were observed not only under overexpression conditions but also under knockdown and knockout conditions, suggesting that TUBL indeed contributes to the regulation of keratinocyte proliferation.

*4. Fig. 3: Some assays to test if RNA structure changes generated in 3B cause deficient TINCR downstream RNA targeting would be needed.*

**[Response]** The functions of TINCR as an RNA were previously shown to include regulation of the stability of a series of mRNAs via the TINCR box as well as sponging of microRNAs such as miR-335, miR-125b, miR-29b, and miR-589-3p (1-6). However, these functions were found to be mediated by the 3'-UTR region of TINCR. Given that the constructs used in **Figure 3** consisted only of the ORF region (261 nt in human), not the full-length TINCR RNA (3733 nt in human), it would be difficult to evaluate the effects of the constructs on downstream RNA targeting in the setting of our experiments.

**References**

1.	Kretz, M., Siprashvili, Z., Chu, C., Webster, D.E., Zehnder, A., Qu, K., Lee, C.S., Flockhart, R.J., Groff, A.F., Chow, J. *et al.* (2013) Control of somatic tissue differentiation by the long non-coding RNA TINCR. *Nature*, **493**, 231-235.

2.	Liu, G.W., Yang, H.J. and Xu, C.H. (2018) Long noncoding RNA TINCR promoted cell proliferation through sponging miR-29b in non-small cell lung cancer. *Int. J. Clin. Exp. Med.*, **11**, 6856-6862.

3.	Dong, H.Y., Hu, J.G., Zou, K.J., Ye, M.L., Chen, Y.W., Wu, C.Y., Chen, X. and Han, M.L. (2019) Activation of LncRNA TINCR by H3K27 acetylation promotes Trastuzumab resistance and epithelial-mesenchymal transition by targeting MicroRNA-125b in breast Cancer. *Mol. Cancer*, **18**, 3.

4.	Li, R., Wang, Y., Xu, Y.X., He, X.L. and Li, Y.L. (2019) Silencing the long noncoding RNA, TINCR, a molecular sponge of miR-335, inhibits the malignant phenotype of epithelial ovarian cancer via FGF2 suppression. *Int. J. Oncol.*, **55**, 1110-1124.

5.	Guo, F.D., Zhu, X.Y., Zhao, Q.Q. and Huang, Q.R. (2020) miR-589-3p sponged by the lncRNA TINCR inhibits the proliferation, migration and invasion and promotes the apoptosis of breast cancer cells by suppressing the Akt pathway via IGF1R. *Int. J. Mol. Med.*, **46**, 989-1002.

6.	Lu, D., Di, S.H., Zhuo, S.S., Zhou, L.Y., Bai, R.M., Ma, T.S., Zou, Z.G., Chen, C.N., Sun, M.M., Tang, J.H. *et al.* (2021) The long noncoding RNA TINCR promotes breast cancer cell proliferation and migration by regulating OAS1. *Cell Death Discov*, **7**, 41.

*5. It will be interesting (the authors may need to discuss this) if the authors can generate a ULCAR specific antibody to demonstrate its tissue distribution, or if its expression changes in human diseases. The former question can be answered by taking advantage of the Flag-knockin mice the authors have generated.*

**[Response]** As suggested by the reviewer, we analyzed the tissue distribution of TUBL among the brain, lung, liver, dermis, and epidermis with the use of *Tubl*^FLAG/FLAG^ mice. TUBL was detected only in the epidermis (**new Figure 1G**). Although TINCR was weakly expressed also in the lung at the RNA level (**Figure 3F**), the extent of TUBL expression in the lung was probably below the detection sensitivity of immunoblot analysis.

Given that the abundance of TINCR RNA has been shown to be up-regulated in human cancers, the expression of TUBL is also likely to be increased. It will be important to generate high-sensitivity antibodies to TUBL so as to investigate how its expression is altered in human tumors. We have now addressed these points in the Results and Discussion sections of the revised manuscript (page 6, lines 143-148 and page 13, lines 338-340).

*6. Given that this is a very novel and exciting study revealing the first protein function of ULCAR, it will be necessary to speculate how ULCAR modulates cell cycle progression? Any specific targets or binding partners of ULCAR could explain its function? Or is this related to function from other type 2 ubiquitin like molecules?*

**[Response]** To explore the molecular function of TUBL, we purified protein complexes containing TUBL from NHEKs and HaCaT cells transiently expressing TUBL-(C)FLAG by immunoprecipitation with antibodies to FLAG. The eluted proteins were subjected to semiquantitative LC-MS/MS analysis, resulting in the identification of several molecules that were specifically associated with TUBL on the basis either that they were detected specifically in at least one TUBL-(C)FLAG replicate or that the average iBAQ (intensity-based absolute quantification) value for the three TUBL-(C)FLAG replicates was >2 times that for the control replicates (**new Figure 7A and new S3 Table**). Gene ontology (GO) term analysis of the identified TUBL binding proteins revealed enrichment of several cell cycle–related terms, such as those involving “ubiquitin-protein ligase activity involved in mitotic cell cycle” (**new Figure 7B**). However, we found that most genes constituting such GO terms actually encoded subunits of the proteasome complex, with many being commonly identified in both HaCaT and NHEK cells (**new Figure 7C and new S4 Table**).

TUBL is a type 2 Ubl protein but does not harbor any other apparent domains. It is therefore a Ubl-only protein that associates with the proteasome. In contrast, RAD23 and DSK2, both of which are representative type 2 Ubl proteins, contain a Ubl domain that associates with the proteasome and a Uba (ubiquitin-associated) domain that interacts with ubiquitylated proteins, allowing them to facilitate the recruitment of ubiquitylated proteins to the proteasome for efficient proteolysis. TUBL is expected to lack such an ability as a result of its lack of a Uba domain, suggesting that TUBL might function as a negative regulator that modifies the interaction between authentic Ubl-Uba proteins and the proteasome. Although the detailed molecular mechanism by which TUBL regulates cell cycle progression remains unknown, we believe that it is beyond the scope of our present study. We have now addressed these issues in the revised manuscript (page 11, lines 282-305).
